# Soft System Based on Fiber Bragg Grating Sensor for Loss of Resistance Detection during Epidural Procedures: In Silico and In Vivo Assessment †

**DOI:** 10.3390/s21165329

**Published:** 2021-08-06

**Authors:** Francesca De Tommasi, Daniela Lo Presti, Francesca Virgili, Carlo Massaroni, Emiliano Schena, Massimiliano Carassiti

**Affiliations:** 1 Unit of Measurements and Biomedical Instrumentation, Università Campus Bio-Medico di Roma, Via Alvaro del Portillo, 00128 Rome, Italy; f.detommasi@unicampus.it (F.D.T.); d.lopresti@unicampus.it (D.L.P.); francesca.virgili@alcampus.it (F.V.); c.massaroni@unicampus.it (C.M.); 2 Unit of Anesthesia, Intensive Care and Pain Management, Università Campus Bio-Medico di Roma, Via Alvaro del Portillo, 00128 Rome, Italy; m.carassiti@unicampus.it

**Keywords:** analgesia, epidural procedure, fiber Bragg grating sensor, instrumented syringe, loss of resistance, soft sensors

## Abstract

Epidural analgesia represents a clinical common practice aiming at pain mitigation. This loco-regional technique is widely used in several applications such as labor, surgery and lower back pain. It involves the injections of anesthetics or analgesics into the epidural space (ES). The ES detection is still demanding and is usually performed by the techniques named loss of resistance (LOR). In this study, we propose a novel soft system (SS) based on one fiber Bragg grating sensor (FBG) embedded in a soft polymeric matrix for LOR detection during the epidural puncture. The SS was designed to allow instrumenting the syringe’s plunger without relevant modifications of the anesthetist’s sensations during the procedure. After the metrological characterization of the SS, we assessed the capability of this solution in detecting LOR by carrying it out in silico and in clinical settings. For both trials, results revealed the capability of the proposed solutions in detecting the LOR and then in recording the force exerted on the plunger.

## 1. Introduction

Among many drug therapies, epidural analgesia is a well-established loco-regional technique widely used in pain relief in applications with relevant clinical and socio-economic impacts [1,2]. It allows achieving a central nerve block by injecting an anesthetic within the epidural space (hereafter ES) and close to the nerve that transports the pain. This technique is used for those facing lower back pain, which represents a relevant global health burden affecting a high percentage of people at least once over the whole of their lives [3,4]. Epidural analgesia is also broadly accepted during labor since it is effective in increasing maternal satisfaction and reducing pain without having effects on neonatal status [5]. In addition, it may be considered a relevant factor in the psychological adjustment of women in the postpartum [6,7].

During this procedure, the anesthetic injection within the ES is achieved by two main steps: (1) inserting a specific needle (i.e., the Tuohy needle) between two lumbar vertebrae and (2) delivering the drug through a catheter positioned in the ES. The ES localization is fundamental to avoid potential complications such as headache, nausea and, in the worst case, the perforation of dura mater [8]. The most popular technique to guide the needle within the ES is Loss of resistance (LOR) which is based on the tactile feedback coming from the syringe plunger: when the anesthesiologist pushes the syringe from the skin to the ES, the needle passes through several different tissues’ layers. The last two layers (i.e., the ligamentum flavum and the ES) have a relevant difference in terms of densities; thus, the anesthesiologist perceives a tactile sensation called LOR when the needle breaches the ligamentum flavum and reaches the ES. Over the last years, both imaging-based techniques (e.g., ultrasound-based techniques, X-ray imaging techniques, near-infrared tracking systems) [9,10,11,12] and instrumented systems able to detect the LOR [13,14,15,16,17,18] have been investigated to guide the needle to the ES. However, a large part of the epidural analgesia procedures is performed blindly; thus, their success is operator-dependent [12].

For years, our group has been involved in research projects focused on developing systems for supporting anesthesiologists in LOR detection. In our first attempts, we instrumented the syringe’s plunger with a piezoresistive force sensor. A custom algorithm was also proposed to detect the LOR and was tested in ex vivo animal models and in clinical settings [19,20,21].

Fiber optic sensors may be particularly powerful alternatives to electrical sensors for the development of tools devoted to ES detection. Indeed, their characteristics of small size, flexibility, and good metrological properties make these sensors especially suitable for many biomedical applications [22]. An emerging class of fiber optic sensors, called fiber Bragg grating sensors (FBGs), are gaining broad acceptance in medical applications [23,24]. This technology has been recently used to estimate the force applied to the spinal needle during lumbar puncture [25] and to monitor the whole epidural procedure by instrumenting either a needle or a plunger using FBG [26,27,28,29].

The overall goal of this study is to propose a method to detect the LOR to support anesthesiologists during epidural procedures. Here, we demonstrated the feasibility of a novel FBG-based system for LOR detection on six patients undergoing epidural analgesia. In Section 2, we reported the design and fabrication of the proposed soft system (SS) based on FBG sensor and the assessment of the SS by showing its main metrological characteristics. In Section 3, we described the experimental protocols employed for the experiments carried out on an epidural simulator and in clinical settings. The results obtained for these two scenarios are reported in the same section to assess the capability of our SS in detecting LOR.

## 2. Soft System for LOR Detection: Principle of Work, Design, Manufacturing and Metrological Assessment

This section is devoted to the description of the proposed system for detecting the LOR during an epidural procedure. Firstly, we focus on the design and the fabrication process of the system. Then, we report a description of the working principle of the SS based on FBG. Lastly, we report the device response to applied force in a scenario mimicking the clinical settings.

### 2.1. Design and Manufacturing

An FBG-based force sensor was designed to fit the top of the syringe plunger (diameter of ~20 mm and thickness of ~2 mm) for detecting the LOR during epidural procedures. A commercial FBG sensor (AtGrating Technologies, Shenzen, China) with a grating length of 10 mm and a λ_B_ of 1536 nm was encapsulated into a soft matrix made of silicone rubber (DragonSkin™ 30, Smooth-On, Macungie, PA, USA). The matrix has a cylindrical shape (diameter of 26.4 mm and thickness of 8 mm) with a fitting hole (diameter of 22.4 mm and thickness of 2 mm) for the manual insertion of the plunger. The hole allows achieving a tight adhesion of the SS to the plunger.

**Figure 1:**
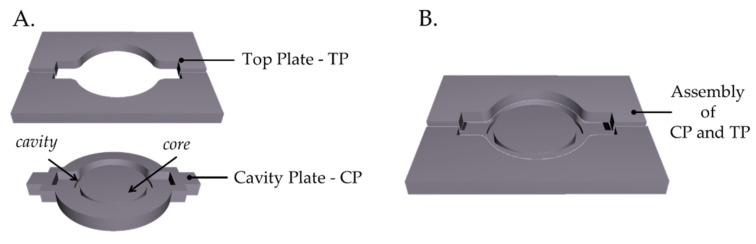
(**A**) CAD of the Top Plate -TP- and the Cavity Plate -CP-, (**B**) their assembly.

A 3D mold was designed in a CAD environment (SolidWorks, Dassault Systems, Waltham, MA, USA) to obtain the cylinder-shaped soft sensor. The mold consists of two main plates (i.e., the Cavity Plate-CP and the Top Plate-TP). The CP has a cylindrical cavity with a core insert to create the fitting hole. The TP consists of two parts designed to allow the FBG encapsulation and easily remove the sensor from the CP. Then, the mold CAD was printed in Polylactic acid–PLA using the 3D printer (Ultimaker S2^+^, Crea3D, Bari, Italy). Figure 1 shows the CAD and the printed CP and SP.

**Figure 2:**
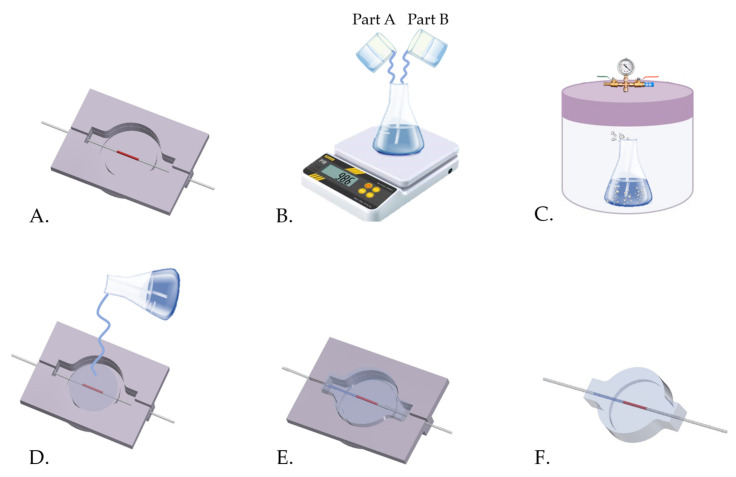
The main manufacturing steps: (**A**) the FBG sensor positioning, (**B**) the silicone components mixing, (**C**) the bubble degassing of the mixture, (**D**) the mixture pouring, (**E**) the silicone curing and (**F**) the soft sensor demolding.

Once the mold was printed, CP and ST were assembled and the FBG sensor placed inside (at one-third of CP thickness), ready to be encapsulated into the soft matrix, made of a bicomponent silicone rubber (i.e., DragonSkin™ 30), as shown in Figure 2A. Then, the two liquid parts (i.e., Part A and Part B) of DragonSkin™ 30 were firstly mixed 1A:1B by weight (Figure 2B) and vacuum degassed (Figure 2C) to eliminate any entrapped air bubble and poured into the 3D-printed mold (Figure 2D). After curing the silicone for 24 h at room temperature (Figure 2E), the soft sensor was demolded (Figure 2F) and arranged to be used for the LOR detection.

### 2.2. Soft System Based on Fiber Bragg Grating Sensor: Working Principle

An FBG is an optical sensor manufactured within the core of an optical fiber in which a variation of the refractive index occurs. This modulation is achieved by lighting the optical fiber core using a spatially variable pattern of intense UV laser light. FBG performs as a pass-band filter. Thus, when a wideband light signal enlightens the fiber, a small portion of wavelengths is reflected while the remaining ones move unperturbed along the fiber. The reflected narrow spectrum is centered around a specific wavelength (i.e., Bragg wavelength λ_B_) specifically of the FBG and expressed by the following equation [30]:

λ_B_ = 2 · n_eff_ · Λ (1)

where n_eff_ is the effective refractive index, and Λ is the grating period. The FBG principle of work is based on the λ_B_ shift (i.e., Δλ_B_) that occurs according to the temperature and strain affecting the grating. Δλ_B_ can be evaluated as follows [31]:

(2) $$\frac{\Delta \lambda_{B}}{\lambda_{B}}\;=\;(1-\;\rho_{\alpha })\;\cdot \;\varepsilon \;+\;(\alpha \;+\xi )\;\cdot \;\Delta T$$

ρ_α_, α and ξ denote the photoelastic, thermal expansion and thermo-optical coefficients, respectively; ε represents the strain and ΔT the temperature variations. In this work, we considered a temperature-free configuration, assuming that the temperature changes are negligible since the thumb presses the device during the whole procedure.

**Figure 3:**
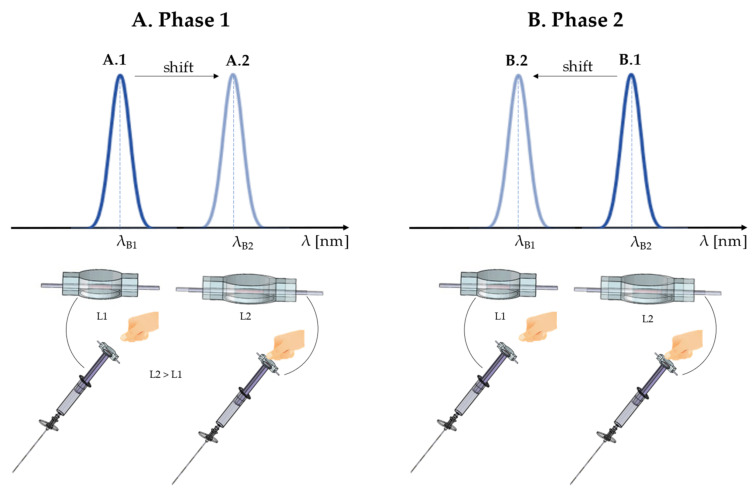
Working principle of our system during the epidural procedure. (**A**) During Phase 1, the clinician presses their thumb on the device. (**B**) During Phase 2, the clinican stops pressing on the plunger.

Figure 3 shows the working principle of our proposed system during the epidural procedure to better clarify how it is possible to detect LOR with an FBG-based technology. Basically, the procedure can be divided into two main phases:

(1) After the correct Tuohy needle’s localization between two lumbar vertebrae, the clinician’s thumb pushes the smart device placed on the plunger, allowing the advancement of the needle through several soft tissue layers up to the ligamentum flavum. Due to the force applied by the finger, the FBG encapsulated within the polymeric matrix is strained. Therefore, the FBG stretches from length L1 to L2, causing an increment of Λ with a consequent λ_B_ increase (i.e., Δλ_B_) from λ_B1_ to λ_B2_ (see Figure 3A).

(2) When the Tuohy needle crosses the ligamentum flavum and reaches the ES, the clinician feels the LOR and stops pushing the syringe plunger. During this phase, the polymeric matrix slackens due to the LOR and the λ_B1_ decreases; then, the clinician stops pushing and the FBG returns to its original condition. Summing up, the FBG moves from the L2 to L1, causing a Λ decrease with a consequent λ_B_ shift from λ_B2_ to λ_B1_ (see Figure 3B).

### 2.3. Metrological Characterization

To assess the response of the proposed system to the force (F), we performed seven compression tests through a testing machine (hereafter TM, model 3365, Instron^®^, Norwood, MA, US). During the tests, an external F ranging between 0 N and 40 N was applied with a compression rate of 3 mm·min^−1^ to reproduce a quasi-static condition. The applied F was measured by a load cell with a full-scale value of 500 N (Serial Number 69376, Instron^®^, Norwood, MA, US) and an accuracy of ±0.25% of the read value.

**Figure 4:**
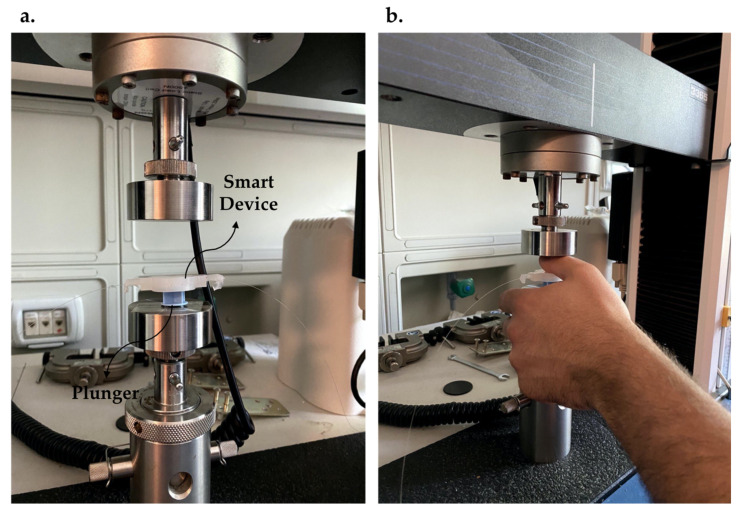
(**a**) Positioning of the plunger and the smart device on the lower plate of the TM. (**b**) Volunteer’s thumb applied on the smart device during the compression tests.

As suggested by [32], the F was applied to the extremity of the syringe plunger, placing the SS above it (see Figure 4a). Between the lower plate of the TM and the SS, a volunteer placed his thumb in the middle of the polymeric matrix to cover the entire sensitive length of the FBG (see Figure 4b). The proposed setting allowed mimicking the real-world scenario during which the clinician pushes the syringe’s plunger using their thumb to advance the Tuohy needle through the different soft tissue layers. The F values recorded by the TM were collected at a frequency of 100 Hz. Simultaneously, the SS was connected to an optical interrogator (si255, Hyperion Platform, Micro Optics Inc., Atlanta, GA, USA) to store λ_B_ values at a sampling frequency of 1 kHz. At the very beginning of the experiment, we asked the volunteer to press on the device with his thumb to clearly identify the compression test’s starting point.

**Figure 5:**
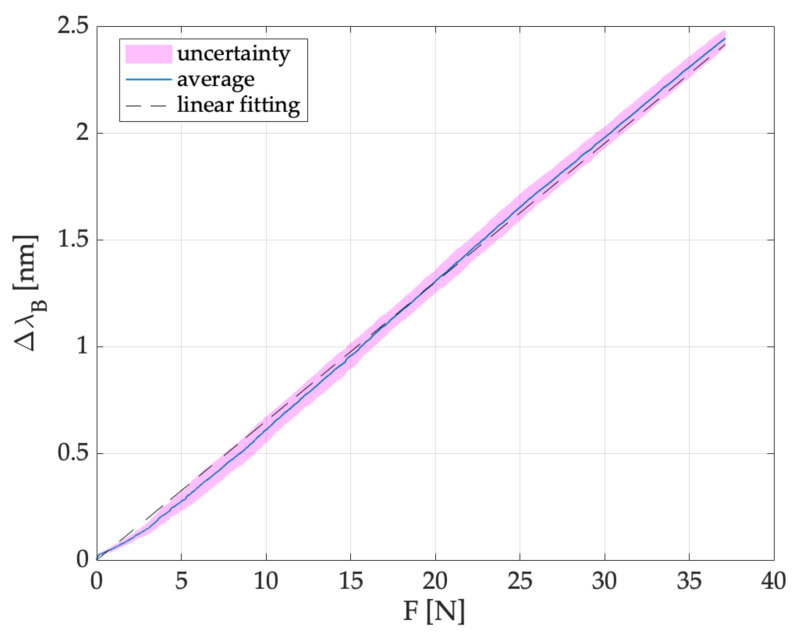
Δλ_B_ vs. F (applied using the TM) of the proposed device. The continuous line in light blue refers to average Δλ_B_ vs. F, the highlighted areas in magenta refer to the uncertainties and the dotted black line shows the linear fitting.

After performing the compression tests, the TM and optical interrogator data were post-processed in MATLAB^®^ environment to obtain the calibration curve (Δλ_B_ vs. F, shown in Figure 5). Firstly, the F and Δλ_B_ data were synchronized considering the minimum value after the peak recorded when the operator applied F on the device. After the synchronization, the signals recorded by the FBG were down-sampled at the frequency of the TM (i.e., 100 Hz, lower than the one of the optical interrogator). Later, we evaluated the averaged Δλ_B_ values and the uncertainty considering the seven compression tests. The t-Student distribution with six degrees of freedom and a confidence level of 95% was considered to estimate the uncertainty. Additionally, a linear fitting model was used to calculate the sensitivity value (i.e., S) of our system to the force applied. Results provided an S value equal to 0.065 nm·N^−1^. The agreement between the linear model and the experimental data is testified by the high R^2^ value (i.e., 0.998), thus revealing a satisfactory fit between theoretical and experimental data.

## 3. Test and Feasibility Assessment of the Soft System for LOR Detection

To evaluate the performance of the SS in detecting the LOR during the epidural procedure, we performed two different trials (i.e., in silico and in vivo). In this section, we describe in detail the experimental set-up employed to perform these experiments and the results obtained.

### 3.1. In Silico Experiments

**Figure 6:**
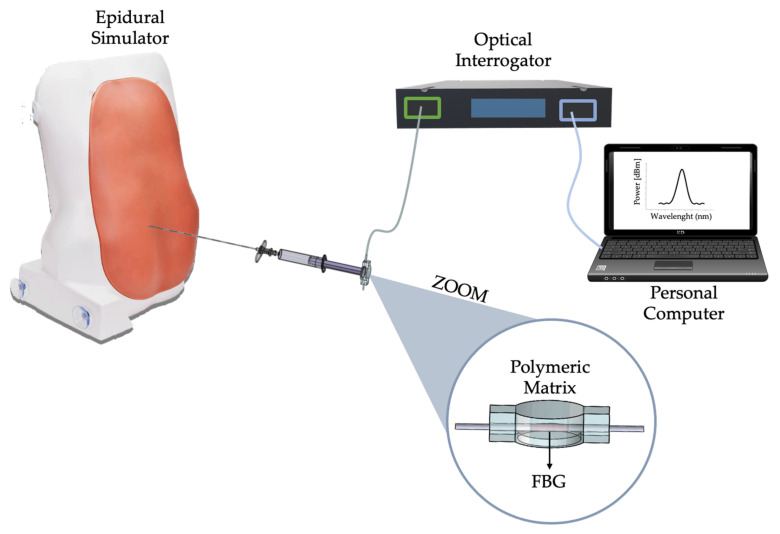
Experimental set-up employed during in silico trials.

To preliminary assess the capability of the SS in detecting LOR, a first attempt envisaged performing epidural injections using a commercially available epidural simulator (P61, 3B Scientific, 3B Italy, Ozzano Bologna, Italy). The simulator was conceived to mimic an anatomical orientation like those of a patient undergoing epidural anesthesia. It is made by different materials that allow reproducing different soft and hard tissues (i.e., skin, subcutaneous adipose tissues, ligamentum flavum, ES, and lumbar spine). Puncture attempts may occur in the simulated intervertebral spaces (from L1-L2 to L4-L5). For these tests, six users performed a total of 25 epidural punctures after receiving training by an expert anesthesiologist (M.C.). The SS was accommodated on the syringe’s plunger, and output of the FBG (λ_B_) encapsulated within the SS was recorded by an optical interrogator (si255, Hyperion Platform, Micro Optics Inc., Atlanta, GA, USA) at a sampling frequency of 1 kHz. The optical interrogator was linked to a personal computer through a LAN cable to display in real-time the λ_B_ changes and simultaneously store data (please refer to Figure 6). To conveniently detect the beginning of the procedure, each user was asked to press three times on the polymeric matrix before performing the puncture.

#### Data Analysis and Results

**Figure 7:**
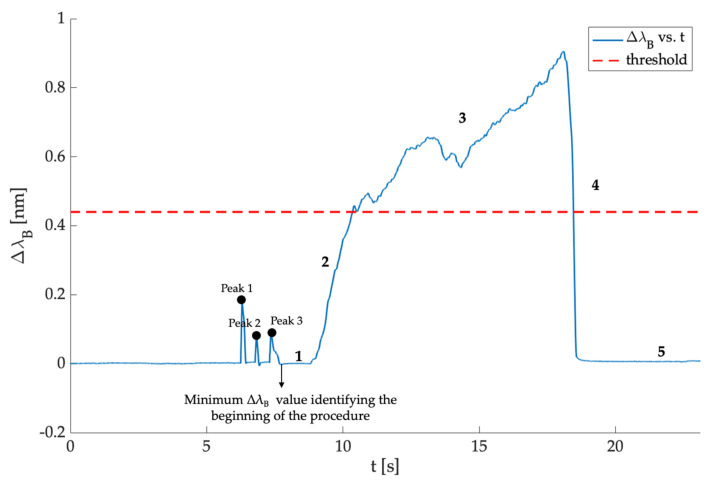
Trend recorded during one of the 25 procedures performed showing: (i) the three peaks related to the force applied three times by the thumb before the procedure, (ii) the minimum Δλ_B_ value identifying the beginning of the procedure and (iii) the five phases labeled in all signals.

After the data collection, λ_B_ values recorded by the SS during each procedure were post-processed in MATLAB^®^ environment to graph the typical trend recorded during the epidural punctures. Firstly, we evaluated the Δλ_B_ for each trial, and then the Δλ_B_ trend as a function of a time was obtained. Subsequently, the resulting signal was cut considering as a starting point the minimum value of Δλ_B_ after the third peak recorded during the phase in which the user pressed three times on the polymeric matrix (as shown in Figure 7).

**Figure 8:**
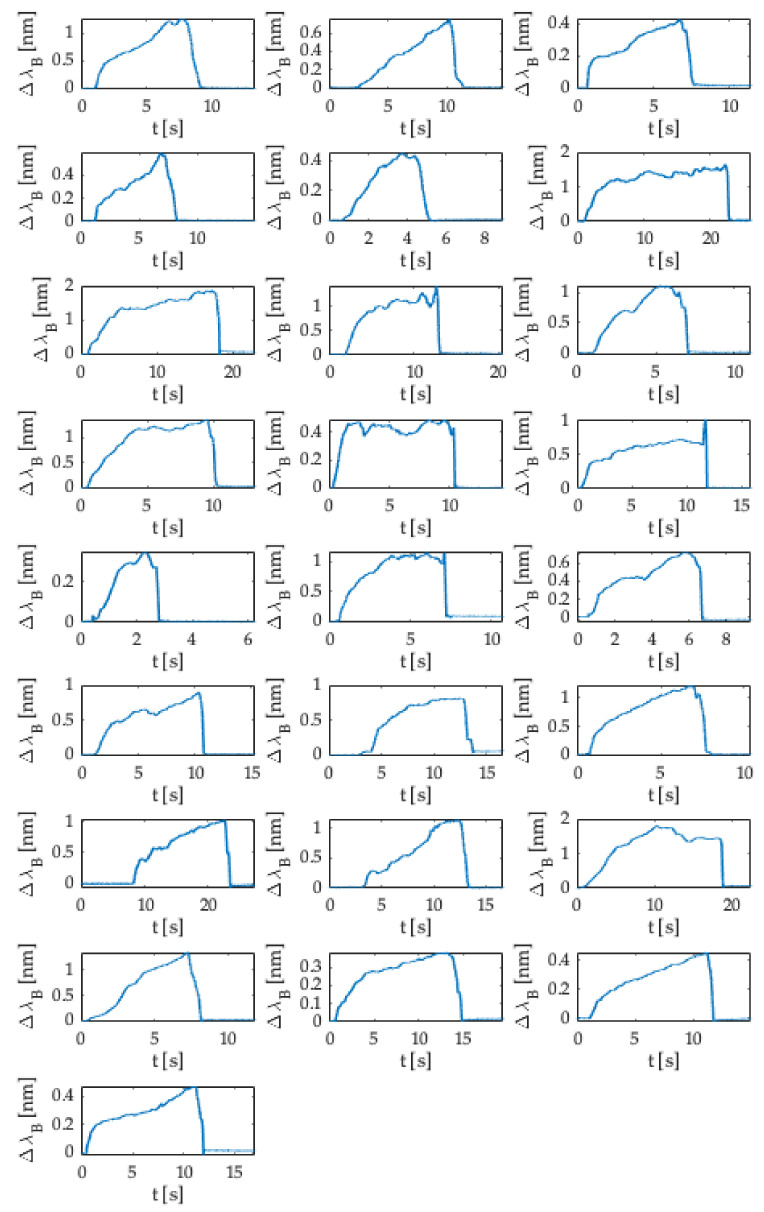
Twenty-five trends (i.e., Δλ_B vs._ t) obtained during the in silico experiments.

Figure 8 shows the 25 trends (i.e., Δλ_B_ (nm) vs. time (s)) matching the executed experiments.

In all the punctures performed, the signals acquired showed approximately the same pattern, which can be categorized into five main phases:

(1) A first phase in which the Δλ_B_ = 0 because the user was not yet pressing on the syringe’s plunger.
(2) A quick increase in Δλ_B_ when the user pressed on the syringe’s plunger.
(3) A phase in which the Δλ_B_ value exceeded a specified threshold when the user pushed the SS to advance the Tuohy needle through the different layers mimicking the anatomical soft tissues. The threshold value of Δλ_B_ was different in each test according to the force applied by the user on the SS.
(4) An abrupt downturn in the value of Δλ_B_ due to the LOR corresponding to the transition from the material mimicking the ligamentum flavum to replacing the ES.
(5) A final phase in which the Δλ_B_ returned to zero at the end of the epidural puncture.

Figure 7 shows one of the trends recorded during the experiments to easily identifying the five phases and the ΔλB threshold. It is worth noting that each procedure has a different duration depending on the time needed by the user to identify the ES.

**Figure 9:**
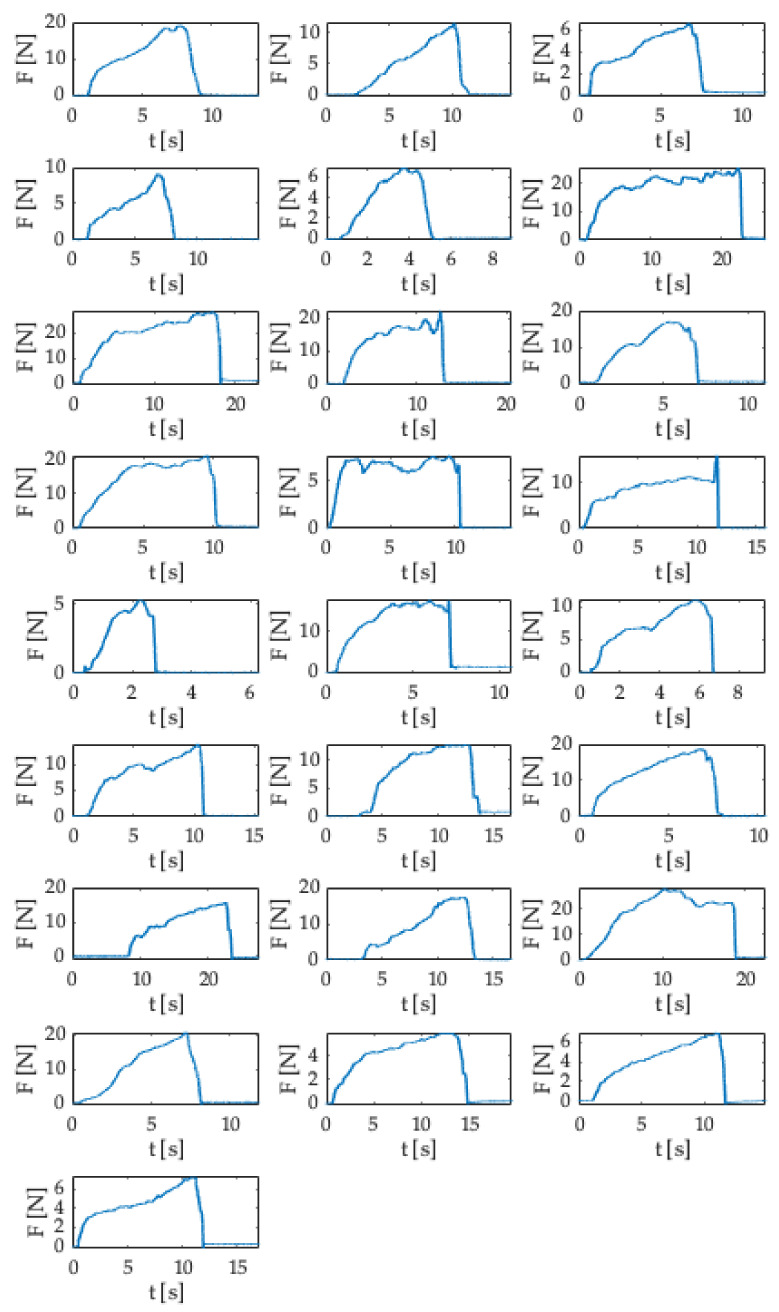
Twenty-five trends (i.e., F (N) _vs._ t (s)) estimated during the in silico experiments.

According to the Δλ_B_ values recorded during the experiments, the force applied to the plunger was estimated for each test. The force was calculated considering the sensitivity estimated by the calibration shown in Figure 5 (i.e., 0.065 nm·N^−1^). Figure 9 shows the force trends as a function of time (i.e., F (N) vs. t (s)) obtained for all the performed experiments. As clearly visible, the maximum force value was recorded at the end of phase 3 (i.e., phase in which the user advanced the Tuohy needle through the different layers of the epidural simulator, please refer to Figure 7) just before the LOR occurred. The maximum values recorded ranged from 6 N to 22 N, according to the complexity experienced by the user in crossing the several materials before reaching the ES.

### 3.2. In Vivo Trials

**Figure 10:**
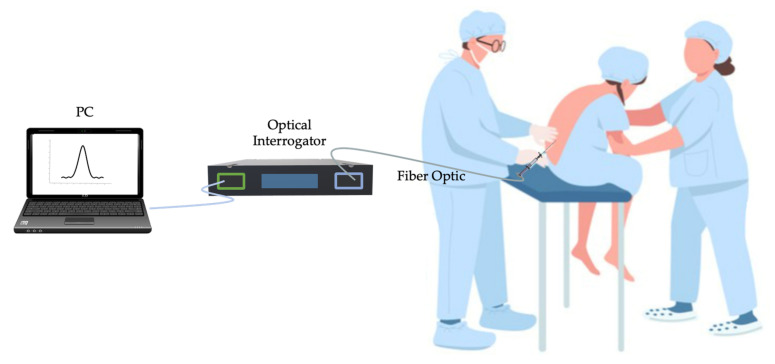
Experimental set-up used during in vivo trials.

After the promising results obtained during the assessment of the SS in silico trial, we tested the capability of the proposed solution in clinical settings. An expert anesthesiologist (i.e., M.C.) with more than 20 years of experience performed epidural injections in six patients affected by either spinal canal stenosis or chronic back pain. All the patients were enrolled in accordance with the Declaration of Helsinki and undersigned the informed consent. The study was approved by the Ethical Committee of University Campus Bio-Medico of Rome (Ref: 04.16-OSS). Each patient was instructed to lie in a fetal position allowing easier identification of the puncture site. Epidural punctures occurred in the intervertebral spaces between either L4-L5 or L5-S1 according to the patient’s pathology. Following the preparation of the sterile field and the insertion of the Tuohy needle in the intervertebral area, the clinician placed the SS on the syringe’s plunger. The SS was connected to the optical interrogator (si255, Hyperion Platform, Micro Optics Inc., Atlanta, GA, USA) to record the FBG’s output, and the LAN connection between the interrogation unit and a PC allowed storing data (see Figure 10). As for in silico experiments, the data collection was carried out with a sampling frequency of 1 kHz. Before performing the procedure, the anesthesiologist was asked to apply a relevant force to the SS to identify the procedure’s starting point.

#### Data Analysis and Results

**Figure 11:**
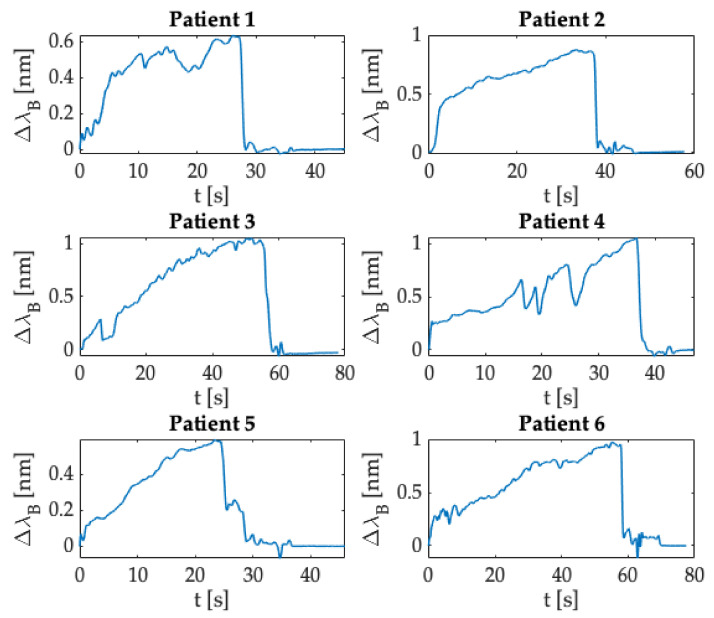
Δλ_B_ collected during the whole procedure in clinical settings.

Figure 11 shows the Δλ_B_ trend as a function of time obtained for each epidural procedure performed by the anesthesiologist in enrolled patients. After collecting data, a custom-made algorithm developed in MATLAB^®^ environment allowed data processing. As described in Section 3.1.1, we evaluated the Δλ_B_ recorded during each procedure.

The procedure’s starting point was identified in correspondence with the Δλ_B_ sample after the peak related to the force applied by the clinician before performing the procedure. Subsequently, data were smoothed with a moving average filter to reduce noise contributions. Compared to the trends obtained for in silico experiments, results found for in vivo trials immediately showed a rapid increase in Δλ_B_ as the anesthesiologist started the procedure promptly after the synchronization peak. This increment was due to the force applied by the clinician to advance the Tuohy needle. Then, Δλ_B_ values exceeded a certain threshold (i.e., Δλ_B_ greater than a specific value expressed in nm), and an abrupt decrease occurred because of the Tuohy needle’s crossing from the ligamentum flavum to the ES. This phase corresponded to the LOR feeling experienced by the clinician during the procedure. Finally, the Δλ_B_ returned to zero at the end of the epidural puncture, when the anesthesiologist did not apply any force to the syringe plunger and thus on the SS.

**Figure 12:**
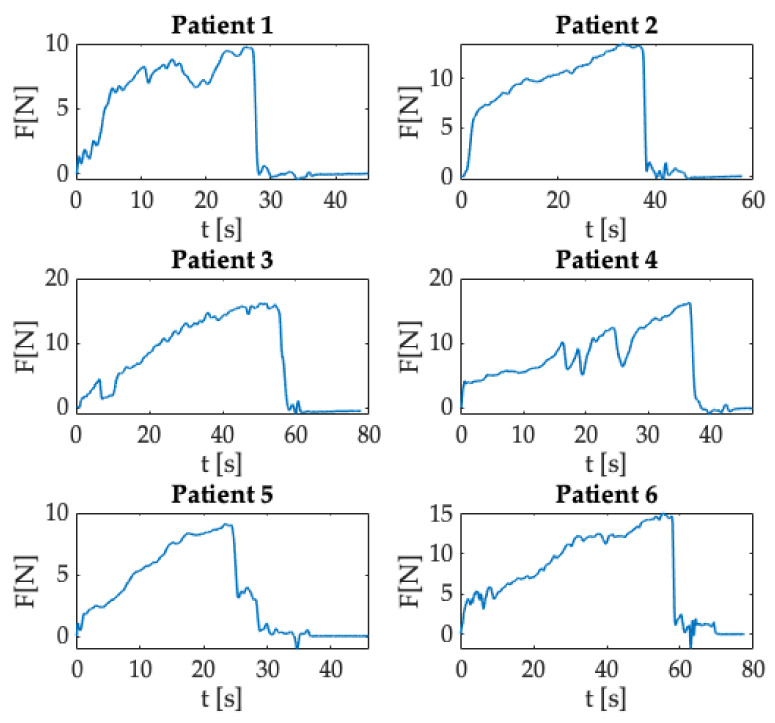
F exerted during the whole procedure in clinical settings.

As shown for the results of in silico experiments (Section 3.1.1), from the Δλ_B_ values recorded for all the trials executed on the patients enrolled, we evaluated the force applied by the clinician during the epidural punctures (please refer to Figure 12). In this case, the maximum force values were in the range of 6 N–15 N.

## 4. Discussions and Conclusions

Epidural analgesia is a broadly practiced technique in pain management [2]. This procedure involves the injection of anesthetics or other pain relievers into the ES. The correct ES identification may be demanding because of its very small size [33]. To date, clinicians perform the procedure by means of the LOR method, which can be accomplished by stuffing the syringe with air or saline solution as preferred by the physician [34]. The LOR feeling is associated with the different density between the ligamentum flavum and the ES. Nevertheless, this method is operator-dependent and does not ensure the right ES identification. Treatment failure can lead to severe patient impairments, including neurological disorders [35]. Therefore, for years, extensive research efforts have focused on the development of smart systems based on different sensing technologies (mainly piezoresistive sensors, and fiber optic sensors) to support clinicians during epidural injections [19,20,21,25,27,28]. In recent decades, FBGs have gained considerable momentum in a wide range of biomedical applications ranging from biomechanics to minimally invasive surgery and physiological monitoring [24,36,37,38,39]. The FBGs’ popularity is mainly ascribed to valuable features such as small size, flexibility, high sensitivity, and fast response time [31].

In this study, we proposed a SS based on FBG-technology for detecting LOR during epidural analgesia. This solution was conceived to be fitted on the syringe’s plunger, avoiding both adding additional tools for carrying out the procedure and altering the traditional clinical settings. We proposed a cylindrical instrumented matrix made of DragonSkin™ 30 silicone rubber embedding an FBG with 1 cm in length. The use of platinum silicone allowed strengthening the optical fiber and simultaneously increase its handling. The desired SS shape was obtained by manufacturing a 3D custom-made mold in which we housed the fiber optic and poured the polymer, thus obtaining the soft sensor. After the system manufacturing, we assessed the sensitivity of the SS to the force applied by mimicking a real clinical scenario, obtaining a satisfactory linear fitting (R^2^ = 0.998) with a static sensitivity value of 0.065 nm·N^−1^. To assess the capability of the proposed solution in detecting LOR, we carried out two different trials (i.e., in silico and in vivo). Both in silico and in vivo experiments revealed the capability of this solution in detecting the LOR. Indeed, trends (Δλ_B vs._ t) obtained for both scenarios clearly showed the same behavior marked by a sudden and abrupt decrease in Δλ_B_ value when the Tuohy needle reached the ES in all the trials performed. Additionally, the metrological assessment allowed estimating the force applied by the user/clinician during the whole procedure. To the best of our knowledge, this is the first study in which an FBG was used to instrument the syringe’s plunger for LOR detection. Other earlier investigations demonstrating the feasibility of employing FBG in this specific scenario proposed instrumenting the Tuohy needle [25,26,27,28]. In [25], the authors developed an FBG device for monitoring force during lumbar puncture. The system consisted of two stainless steel cylinders linked by a bar housing an FBG with 3 mm in length at its center. This instrumented system was designed to fit on the spinal needle. However, the clinician is unable to use the syringe plunger to advance the needle; thus this solution may be not easy to use and may require a training session for the physicians before being used. Furthermore, since it is designed to be fixed on the spinal needle, it is conceivable to cause an obstruction to the drug passage. The system showed a sensitivity of −0.046 nm·N^−1^, slightly lower than the value of the SS proposed in this study (i.e., 0.065 nm·N^−1^). Research works carried out by Carotenuto et al. [26,27] resulted in the development of an instrumented Tuohy needle to measure pressure variations during epidural procedure. The authors proposed a fiber optic embedding one FBG positioned in the proximity of the needle tip. In this case, the optical fiber was fitted inside the needle by means of a custom-made system. It is worth noting that this solution was intended as an alternative to the LOR method and requires changes in the traditional method to perform the procedure. Moreover, as the fiber must be inserted into the needle, rapid re-uses are not guaranteed, and there is a high risk of contaminating the sterile field. Finally, the assessment was only performed through in silico testing, without considering potential concerns that would emerge in clinical settings.

Differently from these previous studies, not only is our system a non-invasive solution, but it does not require the insertion of the sensing element inside the needle. In addition, our system does not require any alteration of the standard settings and clinical practice during the procedure. Therefore, it may be easily reusable as it is not intended to be directly in contact with the injected drug. Indeed, the SS was designed as an additional tool to the LOR method for providing an efficient support to the clinician.

After these promising results, future tests will be performed to deeply assess the performance and the usability of our solution in a larger population of patients affected by musculoskeletal disorders and with different clinicians. Therefore, the small study population was one of the limitations of our study. A further limitation was that all the trials were performed by an expert anesthesiologist; thus we will perform further trials involving clinicians with less expertise. From a technical point of view, further analysis will be devoted to assessing the influence of different materials to manufacture the polymeric matrix; in addition, since the thickness influences SS with this working principle [40], we will investigate the influence of the shape and size of the SS on its metrological properties.

## Data Availability

The data presented in this study are available on request from the corresponding author. The data are not publicly available due to privacy reasons.
